# OnabotulinumtoxinA for chronic migraine during pregnancy: a real world experience on 45 patients

**DOI:** 10.1186/s10194-020-01196-1

**Published:** 2020-10-29

**Authors:** Ho-Tin Wong, Modar Khalil, Fayyaz Ahmed

**Affiliations:** 1 Department of Neurosciences, Hull University Teaching Hospitals, Hull, UK; 2 Hull York Medical School, Hull, UK; 3 Spire Hospitals Hull and East Riding, Hull, UK

**Keywords:** Botox, OnabotulinumtoxinA, Pregnancy, Chronic migraine, Headache

## Abstract

**Objective:**

To report the pregnancy outcomes on patients with chronic migraine exposed to onabotulinumtoxinA from Hull Headache Clinic.

**Background:**

Migraines are common in women of reproductive age and those with chronic migraine have a major impact on their activities of daily living and health-related quality of life. Apart from low dose amitriptyline and beta-blockers all other prophylactic agents have proven teratogenic effects. OnabotulinumtoxinA is approved as preventive treatment for adult patients with chronic migraine, although its impact on pregnancy is unknown.

**Methods:**

We prospectively collected data for efficacy and safety on all patients treated with onabotulinumtoxinA at the Hull Headache Clinic. The toxin is administered as per PREEMPT paradigm. Female patients of reproductive age group receiving onabotulinumtoxinA are given advice on contraception and the unknown impact of the toxin on pregnancy. They are asked to report pregnancy when they are appraised on the risk/benefit of treatment continuation. All patients are consented for access to their medical records and pregnancy outcome and those who wished to continue are asked to sign a disclaimer. Pregnancy outcome data was collected on all patients for the mode of delivery, birth weight and congenital malformation and any other unexpected outcomes.

**Results:**

Over 9 years period 45 patients reported pregnancy while receiving onabotulinumtoxinA. All patients had received onabotulinumtoxinA within 3 months prior to the date of conception. 32 patients wished to continue treatment during pregnancy while the remaining 13 stopped treatment. Apart from 1 miscarriage in the treatment group, all patients had full term healthy babies of normal birth weight and no congenital malformations.

**Conclusion:**

We report our experience of 45 patients exposed to onabotulinumtoxinA during pregnancy. Although the numbers are small, there was no impact of the toxin found on the pregnancy outcomes.

## Introduction

Migraines are common in women of reproductive age and its control may deteriorate although 50–75% of female migraineurs experience a marked improvement during pregnancy with a significant reduction in frequency and intensity of their attacks, if not a complete resolution [1]. Moreover, there is limited evidence demonstrating safety and efficacy of the oral preventative agents in pregnancy with only amitriptyline and low propranolol deemed suitable for use [2]. Topiramate and sodium valproate, on the other hand, have well established teratogenic effects as shown by the pregnancy register in epilepsy [3]. Some women or clinicians may therefore choose to discontinue or avoid preventative medication during this time.

OnabotulinumtoxinA has been the established treatment for chronic migraine in the UK where 3 or more oral preventative agents have failed [4]. Its use in pregnancy has not been fully evaluated with only one case report in the literature [5]. In the current study, we report our experience of 45 pregnant patients receiving onabotulinumtoxinA (Botox) for chronic migraine from a tertiary centre (Hull) in the UK.

## Methods

The current study was performed in a single headache centre (Hull) in the United Kingdom. Subjects were recruited and treated over 9 years between 2010 to 2019. A total of 972 patients received treatment for chronic migraine. 99% (*N* = 957) patients had failed three oral preventive treatment as per NICE (National Institute for Health and Care Excellence) guidelines. 82% (*N* = 797) of the cohort were females.

All patients receiving onabotulinumtoxinA for chronic migraine received prospective follow up and women of reproductive age were informed about the uncertain impact of onabotulinumtoxinA on pregnancy and were given advice on contraception. Patients receiving onabotulinumtoxinA who reported pregnancy were given an informed discussion about the limited understanding of its impact on pregnancy and any teratogenicity that it may cause. Patients who wished to continue were asked to sign a consent form and a disclaimer. All patients signed consent for our access to their medical records for pregnancy outcomes (modes of delivery and fetal abnormalities etc).

Patients continuing onabotulinumtoxinA injections followed the original 12 weekly cycles of injections. Headache diaries were completed before and after treatment as means of monitoring the therapeutic response to onabotulinumtoxinA to determine if the treatment were to continue or stop. All pregnant women, including those who did not wish to continue treatment were followed up 3 monthly with their headache diaries. No alternate prophylaxis was given to those who chose to stop onabotulinumtoxinA.

## Results

**Fig. 1:**
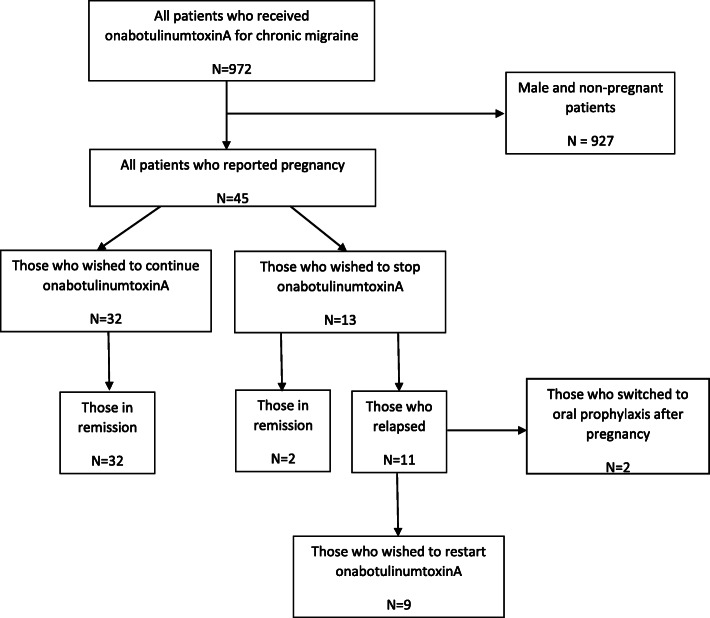
Patient Disposition

During the period 2010–2019, 45 patients were exposed to onabotulinumtoxinA for migraine during pregnancy. The patient disposition is illustrated in Fig. 1. Of the 45 patients who were exposed, 32 patients willingly consented for further injections to continue. The demography (Table 1), number of cycles before pregnancy (Table 2) and possible date of conception in relation to onabotulinumtoxinA therapy (Table 3) is given. All patients were prospectively followed up irrespective of whether they were on onabotulinumtoxin or other treatment. They were followed up for 3.5 years (Range 3 months – 7 years).

**Table 1:** Patient Demographics: Characteristics of pregnant patients exposed to OnabotulinumtoxinA

	All (*N* = 45)	Continue onabotulinumtoxinA (*N* = 32)	Stopped onabotulinumtoxinA (*N* = 13)
Age	30.9 (19–42)	29.8 (19–36)	33.3 (21–42)
Migraine Onset	14.4 (8–23)	14.5 (8–23)	14.2 (8–23)
CM Duration	3.7 (0.5–10)	3.6 (0.5–10)	3.8 (1–8)

**Table 2:** No of cycles received before pregnancy

No of Cycles	All Patients (*N* = 45)	Continued onabotulinumtoxinA (*N* = 32)	Stopped onabotulinumtoxinA (*N* = 13)
1	5	5	0
2	9	5	4
3	9	5	4
4	5	5	0
5	8	6	2
6	3	2	1
7	3	2	1
8	2	1	1
9	1	1	0

**Table 3:** Last injection before conception (weeks)

No of weeks	No of Patients
2	3
3	3
4	4
5	5
6	9
7	7
8	9
9	2
10	3

### Patient demographics

#### Headache outcomes

Of the 32 cases who consented to receive onabotulinumtoxinA continued to show a good treatment response. The response in our cohort was based on the NICE criteria of at least 30% reduction on headache days or a 50% reduction in migraine days as per Hull criteria [6]. Of those who discontinued treatment, 11/13 relapsed and of these, 9/13 consented to go back on treatment after pregnancy whereas 2/13 decided to try oral prophylaxis. Two patients remained in remission during and after pregnancy. Those who relapsed after cessation of treatment showed worsening at 4th to 6th month after the previous treatment when the headache and migraine days reverted back to the pre-treatment level.

#### Pregnancy outcomes

All patients were exposed to onabotulinumtoxinA within 3 months of conceiving. Of the 32 cases consented to continue the treatment during pregnancy, 1 patient had a miscarriage and remaining delivered full term of which there were 2 forceps and 3 caesareans. The two forceps were elected due to previous assisted deliveries. The caesareans were also elective one of which was breach and the other two were planned because of previous difficult vaginal delivery. All 32 newborns were live births with no congenital abnormalities with an average birth weight of 7.3 lbs. (range 5.9–8.2).

##### Patient with miscarriage

This patient who miscarried was 32 years old at the time of pregnancy that ended at the gestational age of 9 weeks and 1 day. She was fit and healthy with no medical problems, a non-smoker who drinks alcohol occasionally and had two previous pregnancies with full term normal vaginal deliveries. There was no obvious cause for the miscarriage. She continued treatment with onabotulinumtoxinA during pregnancy and after miscarriage. She was pregnant again after 4 months and had full term normal vaginal delivery of a healthy baby weighing 8 lbs. She continued onabotulinumtoxinA treatment in the second pregnancy as well.

Those who stopped were all full term deliveries of which there were 1 forceps assisted and two caesareans planned due to difficult previous vaginal deliveries. The average birth weight was 7.5 lbs. (range 6.2–7.8).

## Discussion

OnabotulinumtoxinA in chronic migraine has been approved by the NICE in the UK based on evidence from the PREEMPT studies [7]. However, none of the patients in the original trials were reported to be pregnant and so the efficacy and safety of onabotulinumtoxinA in this population remains unknown. A US survey in 2006 of physicians using onabotulinumtoxinA for all indications found that only 12/396 (3%) had any experience of use in pregnant patients [8]. Of the 12 physicians, 1 declared they were ‘very comfortable’, 5 ‘somewhat comfortable’ and the rest presumably ‘uncomfortable’ with the procedure. Currently in the UK, the advice from the Medicine and Healthcare product Regulatory Agency (MHRA) is that onabotulinumtoxinA should not be used in pregnancy unless clearly necessary’ [9]. Similarly, in the US, the Food and Drug Administration (FDA) lists onabotulinumtoxinA as category C meaning risk is not ruled out but patient benefits may warrant use of the drug in pregnancy [10]. Based on the information and lack of evidence on its harm or safety in the existing literature, we counselled and appraised the patient in detail on the risk-benefit evaluation and choice of other treatment options. The patients were also informed that the typical course of migraine during pregnancy is towards improvement, particularly during the second and third trimester. The discussion was to help them decide if they wished to continue the treatment. Our patients were highly refractory migraineurs with most failing at least three and some up to 6 prior preventive treatments.

In the present study, the 32 patients who remained on treatment with onabotulinumtoxinA through pregnancy continued to show a good response whilst 11/13 who withdrew from treatment showed a relapse in their condition. One patient had a miscarriage (1/32) but the rest had normal vaginal or planned forceps or caesarean deliveries. It is hard to determine the true miscarriage rate in the general population due to reporting bias but the NHS estimated 1 in 8 known pregnancies will miscarry [11] and a 17% miscarriage rate was estimated in one US study [12]. Of the 32 live births, there were no fetal malformations identified, compared to a UK average prevalence rate of 2% [13]. Patients who continued to have onabotulinumtoxinA would have received injections in the second or third trimesters.

Patients in our study were refractory migraineurs with the vast majority tried and failed at least three oral preventive agents with many having tried more than 5–6 class of drugs. Hence, they were keen to continue their treatment with onabotulinumtoxinA in spite of uncertain impact of the drug on pregnancy. None of the patients were overusing painkillers as those with previous excessive analgesic consumption were asked to reduce the intake of painkillers prior to commencing onabotulinumtoxinA and were not on any other concomitant medications.

To our knowledge, there is only 1 case report in the literature of onabotulinumtoxinA treatment for chronic migraine during pregnancy [5]. In this US study, a 26-year-old migraineur was treated with onabotulinumtoxinA but later chose to discontinue this because of the unknown risks in pregnancy. Her migraines worsened but then treatment resumed from 18 weeks gestation with good effect. Fetal movements were noted to be normal throughout and there was no interuterine growth restriction. The delivery was a planned caesarean section at 39 weeks because of breech presentation and the infant had an uneventful birth. The authors reviewed the child at 6.5 years and noted no complications to development.

Safety considerations are of paramount importance for all treatments in pregnancy. OnabotulinumtoxinA exerts its effects by inhibiting nerve terminal exocytosis and by doing so causes neuromuscular blockade. Although there are reports that diffusion of onabotulinumtoxinA after local injection does occur [14], in rabbits, injections of radioactive onabotulinumtoxinA into the eyelids did not lead to spread to distant structures in the body, including the eye [15]. Moreover, onabotulinumtoxinA is a relatively large molecule of 150 kDa and is therefore unlikely to cross the placenta passively, although active transport cannot be excluded [16]. Therefore, at least in theory, one might speculate that onabotulinumtoxinA injections should not have a significant effect on the uterus nor the fetus during pregnancy.

The challenge in conducting trials in pregnancy means that evidence is commonly derived from animal studies or observational studies at best. Animal studies have demonstrated that onabotulinumtoxinA injections are associated with reduced fetal body weight and skeletal ossification as well as abortions, early deliveries and maternal deaths [17]. Such findings have not been confirmed in humans. A 24-year retrospective study of the Allergan global safety database reviewed 574 onabotulinumtoxinA treatments in pregnancy [18]. Overall the prevalence of fetal defects was comparable in both the toxin treated and general populations. It is important to note that the toxin treated group was a heterogeneous population, with a large variety of indications and therefore differences in the sites and doses of toxins injected. This relatively large study did include 22 patients treated for migraine, however the specific outcomes of these pregnancies were not stated and overall results came from pooled data across the different indications [18]. It is also worthy to note that 96% of all cases exposed to onabotulinumtoxinA in this study occurred either prior to conception or during the first trimester and so the effects of injections in the second and third trimester remain largely unknown. Brin et al. in the Allergan study also noted some cases of maternal botulism in the literature which did not report adverse effects on the pregnancy nor the fetus despite significant clinical neuromuscular weakness in the mother [19–21].

## Conclusion

The current study reports our experience of onabotulinumtoxinA for chronic migraine in 45 pregnancies. There were no significant adverse effects in our cohort of patients. However, the numbers are small to draw a conclusion of safety and therefore, it is important to set up a toxin pregnancy register like the one in epilepsy. A similar foreseeable challenge is the evaluation of onabotulinumtoxinA injections in lactation, particularly if more patients consent to have treatment through their pregnancies in the future.

## Data Availability

The datasets used and/or analysed during the current study are available from the corresponding author on reasonable request.

## Abbreviations

FDA: Food and drug administration
MHRA: Medicine and healthcare product regulatory agency
NHS: National health service
NICE: National institute for health and care excellence
